# Growth, phytochemical, and phytohormonal responses of basil to different light durations and intensities under constant daily light integral

**DOI:** 10.1186/s12870-024-05637-w

**Published:** 2024-10-09

**Authors:** Elyas Eghbal, Sasan Aliniaeifard, Mahboobeh Zare Mehrjerdi, Sahar Abdi, Seyedeh Batool Hassani, Tina Rassaie, Nazim S. Gruda

**Affiliations:** 1 Photosynthesis Laboratory, Department of Horticulture, Faculty of Agricultural Technology (Aburaihan), University of Tehran, Pakdasht, Tehran, Iran; 2 Controlled Environment Agriculture Center (CEAC), College of Agriculture and Natural Resources, Faculty of Agricultural Technology (Aburaihan), University of Tehran, Pakdasht, Tehran, Iran; 3 Department of Plant Sciences and Biotechnology, Faculty of Life Sciences and Biotechnology, Shahid Beheshti University, Tehran, Iran; 4 Department of Horticultural Science, INRES-Institute of Crop Science and Resource Conservation, University of Bonn, 53121 Bonn, Germany

**Keywords:** Controlled environment agriculture, Far-red light, Light quality, *Ocimum basilicum*, Phytochrome

## Abstract

Horticulture in controlled environments has been increasingly used to tackle limitations on crop production. As a crucial environmental factor, light regulate plant growth and metabolism. In the present study, basil plants were subjected to different light durations and intensities considering constant daily light integral (DLI). The lighting environment included 200, 300, and 400 µmol m^− 2^ s^− 1^ intensities for 18, 12, and 9 h, respectively. DLI amounted to 12.96 mol m^− 2^ d^− 1^ among all light treatments (LI200 for 18 h, LI300 for 12 h, and LI400 for 9 h). Half of the plants under each light treatment were exposed to 30 µmol m^− 2^ s^− 1^ of far-red light. The results indicated the general negative impact of LI400/9 on the growth of basils. Exposure to far-red light hurt the growth of the shoot, while it enhanced stem and petiole elongation. This effect was due to higher gibberellin accumulation, which resulted in shade avoidance responses. Exposure to far-red light also reduced anthocyanin and flavonoid contents, as two important nutritional components. Soluble carbohydrates increased, while storage carbohydrates decreased by increasing lighting duration/decreasing light intensity or by far-red light inclusion. The lowest antioxidant activity was detected in LI400/9. In the LI200/18, the highest level of auxin and the lowest level of cytokinin were detected, while the LI300/12 exhibited the highest level of gibberellin hormone. Low light intensity and long photoperiod enhanced plant biomass and phytochemical production and are recommended for basil production in controlled environments.

## Introduction

Basil (*Ocimum basilicum* L.) is a vegetable and aromatic herb extensively cultivated for desirable culinary uses. As a member of the Lamiaceae family, basil is renowned for its unique sensory attributes, rendering it a favored ingredient for fresh and cooked applications in numerous cuisines [1]. Basil production in controlled environment agriculture (CEA) has recently attracted much attention. The growth and productivity of basil in CEA are subject to multiple environmental factors, with light emerging as the most critical determinant [2, 3]. Light is also considered the most expensive environmental factor in the CEA, especially for indoor farming practices [4].

Light is an energy source for photosynthesis and a fundamental plant growth and development regulator. It is pivotal in diverse physiological processes encompassing photosynthesis, seed germination, stem elongation, leaf expansion, flowering, and the synthesis of secondary metabolites [5, 6]. Photoreceptors perceive and respond to specific light wavelengths in plants, initiating molecular and physiological responses [7, 8]. Among the various wavelengths within the visible light spectrum, red light (R; 600–700 nm) and far-red (Fr; 700–800 nm) lights have been identified as significant plant growth and development regulators [2, 3]. The Fr light influences photomorphogenesis, which refers to plants’ adaptive responses to light stimuli [9]. Phytochromes, which serve as R and Fr light receptors in plants, capture and transmit light signals, thereby modulating plant morphology, including stem elongation, leaf expansion, and branching [10–12]. When the Fr is present in artificial lighting of indoor farming, the ratio between R and Fr is essential to prevent the occurrence of shade avoidance syndrome (SAS), a morphological disorder leading to the elongation of plant stem and leaf petioles [9]. There is a contradiction in reports regarding the primary driver of SAS in plant species. Low light intensities intensified the SAS effects with Fr [9]. The phytochrome photostationary state (PSS) is an index that shows the impact of artificial lighting on the occurrence of SAS. It has been reported that PSS is a better biological index than the R/Fr ratio to estimate the SAS level, while the R/Fr ratio has been used as a good index for predicting the PSS in the past [13]. Inside the crop canopies, the R/Fr ratio perceived by plant organs varies based on the time and depth of the canopy, in a range within which a slight change in the R/Fr ratio causes a considerable alteration in PSS [9]. Therefore, the R/Fr ratio can be considered the most critical factor influencing the plants’ SAS response because it is the primary driver of PSS. Moreover, R and Fr lights have been observed to impact the biosynthesis of secondary metabolites, notably essential oils, which contribute to the distinct aroma and medicinal properties [13]. In addition to light’s spectral composition, light’s intensity also profoundly affects plant growth and development. Light intensity influences photosynthetic rates, stomatal conductance [14], transpiration [15], and plant biomass accumulation. Optimal light intensity requirements vary among plant species, and deviations from the appropriate range can lead to suboptimal growth and reduced photosynthetic efficiency.

The physiological processes of plants are intricately influenced by light intensity, primarily due to its role in providing energy for photosynthesis. The rate of photosynthesis is directly affected by light intensity, with higher intensities generally resulting in increased photosynthetic rates up to a certain saturation point [16]. As a result of its impact on photosynthesis, light intensity also plays direct or indirect roles in the biosynthesis of primary and secondary metabolites [17, 18]. These metabolites are essential in determining the nutritional quality of plant defense against herbivores, pathogens, and environmental stresses. Light also influences hormone regulation in plants, as hormones are pivotal chemical messengers that govern plant growth, development, and responses to environmental stimuli [19, 20]. Phytohormones such as auxins, gibberellins, cytokinins, and others are involved in various physiological processes, including cell elongation, cell division, and shoot and root development [21]. The relationship between light and hormonal regulation is complex and interconnected. Light signals can influence hormone biosynthesis, transport, and signaling pathways, impacting plant growth and development [12, 19, 20]. For instance, auxins play a role in phototropism and photomorphogenesis, which are light-induced processes that regulate plant orientation and morphology in response to light stimuli [22]. Cytokinins are involved in cell division and shoot development [23], and gibberellins are involved in stem elongation and shade avoidance response when light intensity is limited or there is competition for light when dense canopies are present [24].

The Daily Light Integral (DLI) quantifies the total photosynthetically active radiation (PAR) received by plants over 24 h (mol m^− 2^ d^− 1^). It significantly influences plant growth, morphology, architecture, development, photosynthesis, biomass, yield, and secondary metabolism [25]. Accordingly, the optimal DLI range varies among plant species and cultivars [26, 27]. Factors like plant stage, growth environment, and light quality also interact with DLI and influence plant responses [6]. Therefore, understanding the specific DLI requirements for different plants is essential for maximizing their growth and productivity.

Given the acknowledged impact of light on various facets of plant biology, including growth, development, secondary metabolite biosynthesis, and its potential interactions with hormone signalling pathways, this study sought to explore the influence of light duration and intensity on basil plants. Although different light intensities and durations have been used for growing plants in CEA in previous studies, changes in light intensity or duration impose alterations in cumulative light intensity (Daily Light Integral, DLI). Therefore, in the present study, recognizing that changes in light duration and intensity can result in variations in DLI, we manipulated the lighting conditions to maintain a consistent DLI across different light strategies. We conducted a carefully controlled experiment to explore our hypothesis using light-emitting diodes (LEDs) that emitted specific R and blue (B) wavelengths. Our previous research indicated that 70% R and 30% B light provides the best light quality for basil plants [28]. Therefore, in a controlled system, basil plants were subjected to a fixed R and B (70%:30% ratio) lighting environment with different light intensities and durations while a constant DLI, both with and without Fr exposure.

## Materials and methods

### Plant materials and growth condition

Green basil seeds (‘Mobarake’ ecotype), obtained from a commercial seed company (Pakan Bazr Isfahan, Isfahan, Iran), were sown in 70-cell cultivation trays filled with a substrate containing a 1:1 ratio of coir pith and perlite. After germination and seed sprouting, they were fed with half the strength of Hoagland’s nutrient solution. After four weeks of plant growth, seedlings with three to four true leaves were transplanted into pots (12.5 wide× 10.5 height× 10.5 cm bottom) containing a 2:1 ratio of coir pith and perlite. Then, they were subjected to different light treatments in 18 chambers (six treatments repeated in three blocks) equipped with LED light modules (Parcham Company, Pakdasht, Tehran, Iran). Following transplantation, regular irrigation using the full strength of Hoagland’s nutrient solution was used. The average temperature of 25 ± 2 °C and relative humidity of 50 ± 5% were kept in the growth chambers. Plant sampling was performed after the treatments were applied for 25 days, and morphological, phytochemical, and phytohormonal measurements were conducted on the samples gathered on the last day of plant growth.

### Lighting treatments

**Fig. 1:**
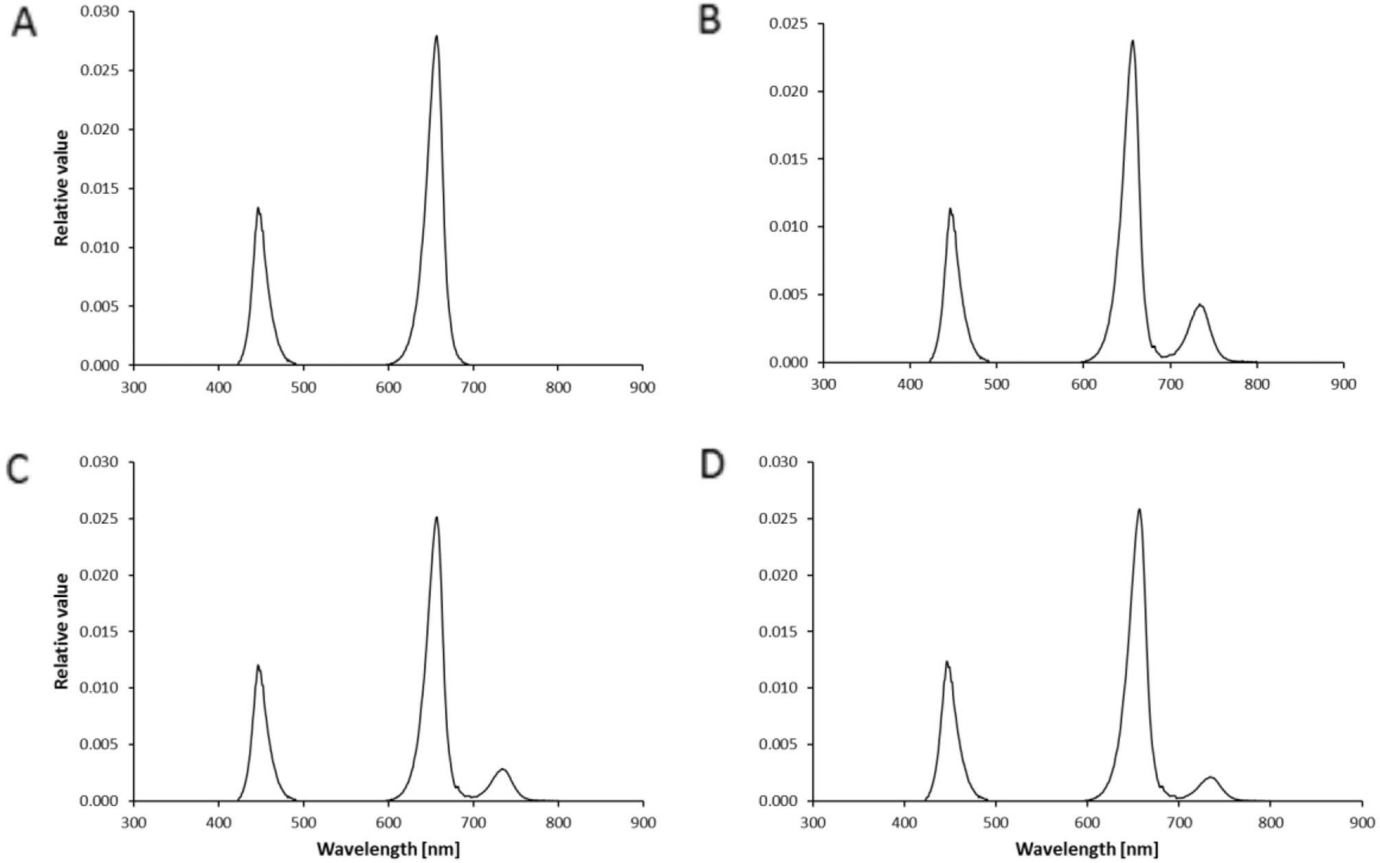
Spectral composition of different lighting environments including light environment with 70%:30% of red (R): blue (B) light without far red (Fr) light (**A**, which was fixed among lighting treatments without Fr light) and for 200 (**B**), 300 (**C**), and 400 (**D**) µmol m^− 2^ s^− 1^ of 70%:30% of R: B light with 30 µmol m^− 2^ s^− 1^ of Fr light

A factorial experimental design was implemented as a randomized complete block design to evaluate the interactions between treatments. The treatments included a light intensity of 200 µmol m^− 2^ s^− 1^ with 18 h of light without [LI200/18] and with Fr light [LI200/18 + Fr], a light intensity of 300 µmol m^− 2^ s^− 1^ with 12 h of light without [LI300/12] and with Fr light [LI300/12 + Fr], and a light intensity of 400 µmol m^− 2^ s^− 1^ with 9 h of light without [LI400/9] and with Fr light [LI400/9 + Fr]. In this study, a fixed R: B ratio 70:30 was applied in the plant cultivation environment [28]. The peak wavelengths were 455, 660, and 730 nm for the B, R, and Fr lights. Light intensities and spectra were monitored using a Sekonic light meter (Sekonic C-7000, Japan), and a timer was employed to determine the duration of the photoperiod. The intensity of Fr light was 30 µmol m^− 2^ s^− 1^ and was utilized simultaneously as the R: B growing light. The spectral composition of different light treatments is presented in Fig. 1. The PSS was calculated based on the method described by Sager et al. [29], which was 0.88 for the R: B lights and 0.82, 0.84, and 0.85, where Fr was also included in LI200/18, LI300/12, and LI400/9, respectively. The R/Fr ratio was 4.4, 6.9, and 9.5 for LI200/18 + Fr, LI300/12 + Fr, and LI400/9 + Fr, respectively. In this experiment, the DLI for the growing light (R: B) was maintained at the same level (12.96 mol m^− 2^ d^− 1^) for all the lighting treatments to ensure consistent daily light availability for all plants.

### Growth and morphological measurements

Morphological traits and growth parameters were measured on the harvested samples. To record the fresh weight of shoots and roots, the two parts of the plant were separated from the stem base and placed on a digital scale with an accuracy of 0.1 g. Plant samples were first placed in paper bags to measure the dry weight of shoots and roots and then dried in an oven at 72 °C for 48 h. After complete drying, they were weighed again using a scale. A ruler with a precision of 1 mm was used to measure plant height and petiole length. The stem diameter was measured using calipers.

To determine the leaf area, the leaves were separated from the plants and placed on a flat white surface with a defined scale. Then, they were photographed, and the leaf area was measured using Digimizer image analysis software (MedCalc Software Ltd, version 5.4. Belgium). These measurements were conducted to assess the morphological characteristics and growth of the plant samples.

### Evaluation of soluble and storage carbohydrates

For this purpose, 500 mg of young, fully developed leaves were collected from each replicate per treatment and ground in liquid nitrogen. They were then mixed with 7 mL of 70% ethanol (w/v) for 5 min on ice and centrifuged at 6700 g for 10 min at 4 °C. After adding 200 mL of the supernatant to 1 mL of an anthrone solution, the absorbance was spectrophotometrically recorded at 625 nm. Standard curve plotting was performed using different concentrations of glucose monohydrate [30].

The precipitates related to soluble carbohydrates from the experiment were dried at room temperature, and starch was solubilized using 52% perchloric acid. Starch was colorimetrically determined using a spectrophotometer at a wavelength of 630 nm [31].

### Phytochemical evaluations

Extracts were prepared from plant samples to measure total phenols, flavonoids, and antioxidant capacity. For this purpose, 1 g of plant sample powder was mixed with 10 mL of 80% methanol and placed in a shaker incubator at 24 °C for 24 h. Then, the samples were centrifuged at 4000 rpm for 15 min, and the supernatant was separated as the extract for evaluating the mentioned compounds and used accordingly.

To evaluate total phenols, the Folin-Ciocalteu reagent was used. Absorption of the samples was spectrophotometrically measured at a wavelength of 765 nm. Concentrations of 100, 200, 300, 400, 500, and 600 mg mL^− 1^ of gallic acid were used to plot the standard curve [32]. To evaluate the concentration of flavonoids, the samples were read at a wavelength of 415 nm, and different concentrations of quercetin were used to plot the standard curve. The percentage of DPPH radical inhibition was calculated by measuring the antioxidant capacity at a wavelength of 515 nm [32]. The solution’s absorbance was measured at wavelengths 530 and 657 nm for anthocyanins measurements [33].

### Measurement of plant endogenous hormones

To measure the level of gibberellins, 0.5 g of leaf tissue obtained from plants exposed to different lighting recipes was macerated in 2.5 mL of ethanol. The obtained extract was read at a wavelength of 254 nm using a spectrophotometer. A standard curve was prepared using specific concentrations of gibberellins to determine the concentration of gibberellins [34].

The method described by Unyayar et al. (1996) measured the level of cytokinins. For this purpose, 0.5 g of plant tissue was weighed and mixed with 15 mL of an extraction solution of methanol, chloroform, and ammonium hydroxide (5:12:3) in a mortar. Then, 6 mL of distilled water was added to the resulting homogenous solution, and the chloroform phase was transferred to a petri dish with a diameter of 15 cm and placed on a heater for complete evaporation of methanol. The spectrophotometer was set to a wavelength of 260 nm for measurement. The values obtained for cytokinins were recorded in µg g^− 1^ of fresh weight using a standard curve prepared for cytokinins [35].

Suzuki et al. (2003) method was used with some modifications for endogenous auxin concentration. The samples’ absorption was read at 530 nm using a spectrophotometer to do so. The amount of IAA in the samples was calculated using a standard curve of zero to 40 mg L^− 1^ of IAA [36].

### Statistical analysis

The experiment was carried out using a factorial design based on a completely randomized block design with three replications. All data were subjected to two-way factorial ANOVA (3*2). The factors include light intensity/duration in three levels [LI200/18, LI300/12, and LI400/9], considering a constant DLI, and Fr light in two levels (with and without Fr light). In each replication, ten plants were grown. For each replication, two plants were used (six plants per treatment). Data analysis was performed using Excel and SAS (v. 9.4, SAS Institute Inc., Cary, NC, USA). Mean comparisons were conducted using Duncan’s test at a significance level of 0.05. Correlations were done by Pearson correlation coefficient using SPSS software (SPSS Inc., Chicago, USA). The p-values were derived from the Pearson correlation analysis, with a significance level set at *P* ≤ 0.05 for the statistical analysis.

## Results

### Growth characteristics of basil plants influenced by light duration and intensity

**Fig. 2:**
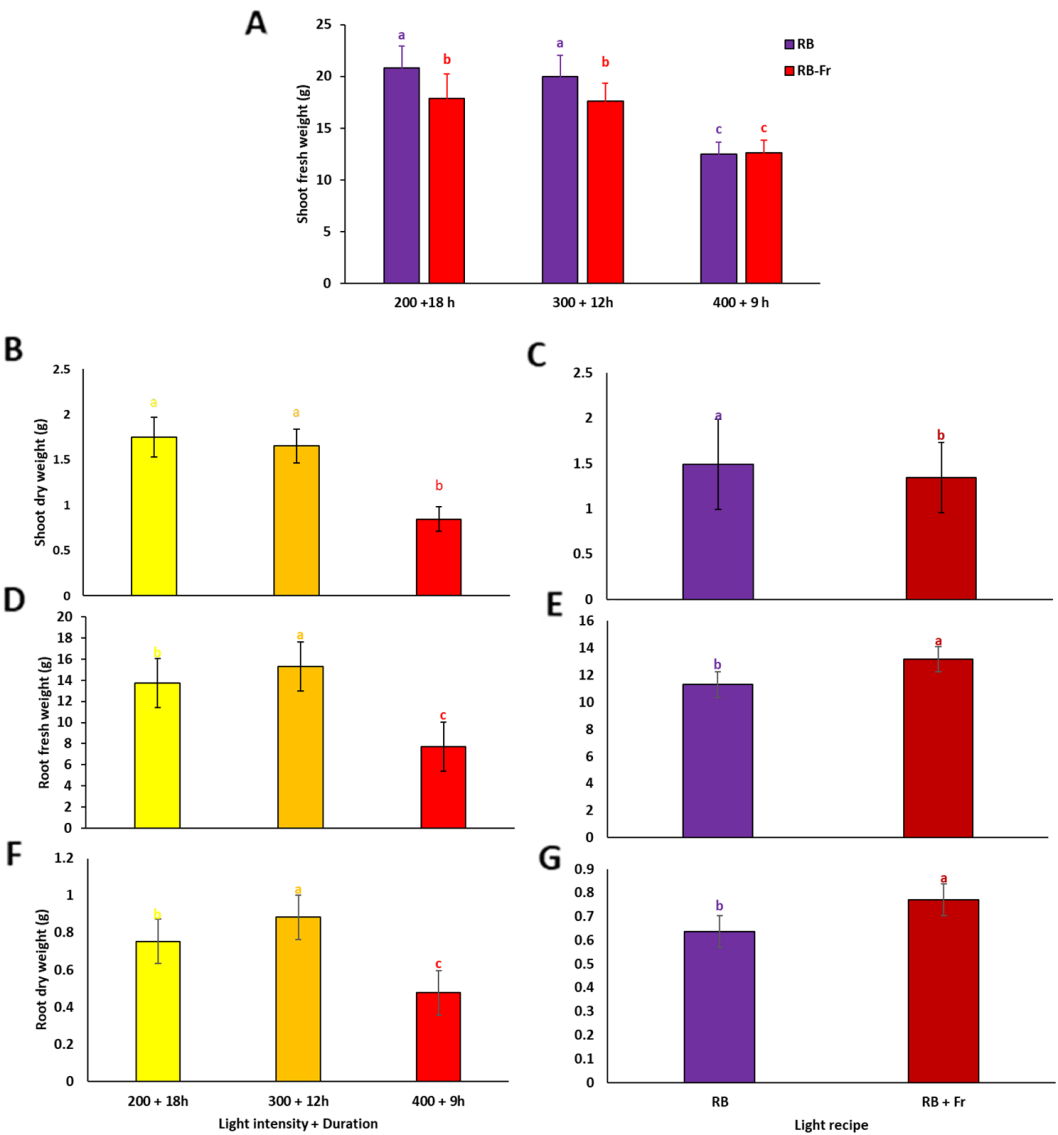
Shoot fresh weight (**A**), shoot dry weight (**B**, **C**), root fresh weight (**D**, **E**), and root dry weight (**F**, **G**) of basil plants grown for 25 days at different lighting environments including light intensity of 200, 300, and 400 µmol m^− 2^ s^− 1^ of 70%:30% of red (R): blue (B) light with durations of 18, 12, and 9 h (200 + 18 h, 300 + 12 h, and 400 + 9 h), respectively, resulted in a fixed daily light integral (12.96 mol m^− 2^ d^− 1^) among all light treatment in an indoor controlled environment with (RB + Fr) or without (RB) far red (Fr) light. Columns are the mean value of three replications. Means with the same letters within the groups are not significantly different (*P < 0.05*)

The results of the variance analysis indicated significant interactions between light intensity/duration and Fr light concerning the shoot fresh weight and leaf area of basil plants. However, no significant interactions were observed for shoot dry weight, root fresh and dry weight, plant height, and petiole length. Plants exposed to LI200/18 h without Fr exhibited the highest shoot fresh weight. However, they were not statistically different from those under LI300/12 without Fr. Conversely, plants exposed to LI400/9 with and without Fr light displayed the lowest shoot fresh weight. Remarkably, the inclusion of Fr light in LI200 (18 h) and LI300/12 treatments negatively affected shoot fresh weight (Fig. 2A). There was a decrease in shoot dry weight with an increase in light intensity and a reduction in light duration. Plants exposed to LI200/18 demonstrated the highest shoot dry weight, showing no significant difference from those under LI300/12 treatment. The lowest shoot dry weight belonged to plants under LI400/9 treatment (Fig. 2B). Plants exposed to a lighting environment incorporating Fr light had lower shoot dry weight than those that did not receive Fr light (Fig. 2C). Under LI300/12 treatment, plants had the highest root fresh weight, demonstrating a significant difference compared to plants under other lighting environments. However, LI400/9 and LI200/18 restricted root fresh weight compared to those under LI300/12 (Fig. 2D). Plants exposed to a lighting environment incorporating Fr light had higher root fresh and dry weight than those that did not receive Fr light (Fig. 2E, G). Plants exposed to LI300/12 displayed the highest root dry weight, while those under LI400/9 represented the lowest root dry weight (Fig. 2F).

**Fig. 3:**
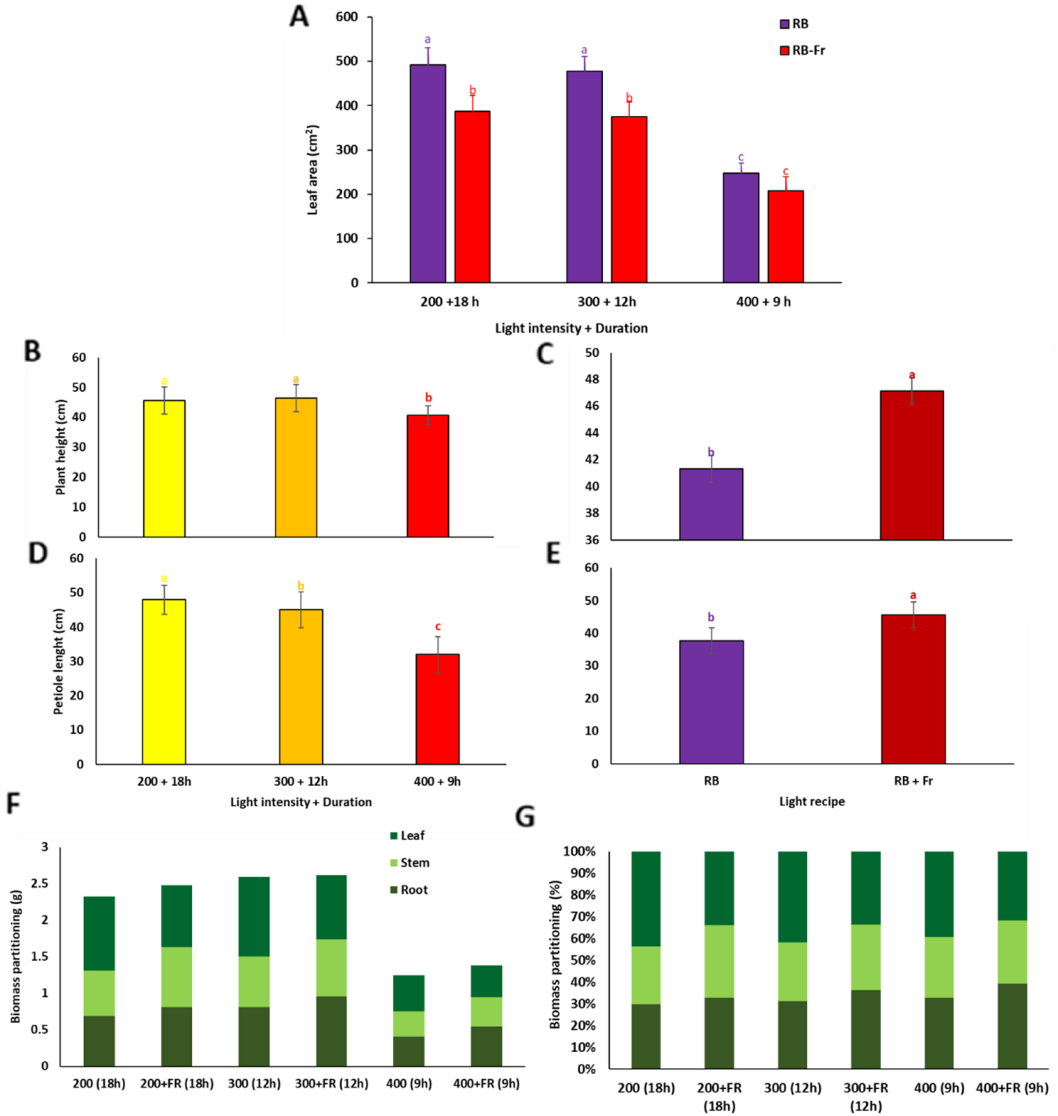
Leaf area (**A**), plant height (**B**, **C**), petiole length (**D**, **E**), biomass partitioning in gram (**F**), and percentage (**G**) of different organs of basil plants grown for 25 days at different lighting environments, including light intensity of 200, 300, and 400 µ mol m^− 2^ s^− 1^ of 70%:30% of red (R): blue (B) light with durations of 18, 12, and 9 h (200 + 18 h, 300 + 12 h, and 400 + 9 h), respectively, resulted in a fixed daily light integral (12.96 mol m^− 2^ d^− 1^) among all light treatment in an indoor controlled environment with (RB + Fr) or without (RB) far red (Fr). Columns are the mean value of three replications. Means with the same letters within the groups are not significantly different (*P < 0.05*)

A positive influence of extended light duration with lower light intensity [LI200/18] without Fr was detected on the leaf area. In comparison, the inclusion of Fr light negatively impacted leaf area in LI200/18 and LI300/12 (Fig. 3A). Plants exposed to LI400/9 had the lowest leaf area among the lighting recipes. Longer light durations with lower intensities (LI200/18) positively affected plant height and petiole length (Fig. 3B, D). Including Fr in all light recipes caused taller plants with longer petioles to emerge (Fig. 3C, E). The shortest plants and petioles were observed in plants exposed to LI400/9.

Partitioning of biomass to different organs (based on the day weight of each organ) was influenced by the lighting strategies (Fig. 3F, G). There was a considerable decrease in biomass partitioned to various organs as the consequence of growing plants under LI400/9 with or without Fr exposure (Fig. 3F). However, when the percentage of biomass allocated to each organ was studied, there was not massive difference among lighting treatments. The only difference was detected in percentages of biomass allocated to the stem of plants exposed to Fr light (Fig. 3G).

### Soluble carbohydrate levels showed an inverse trend in response to light intensity and duration as well as to far red with storage carbohydrate levels

**Fig. 4:**
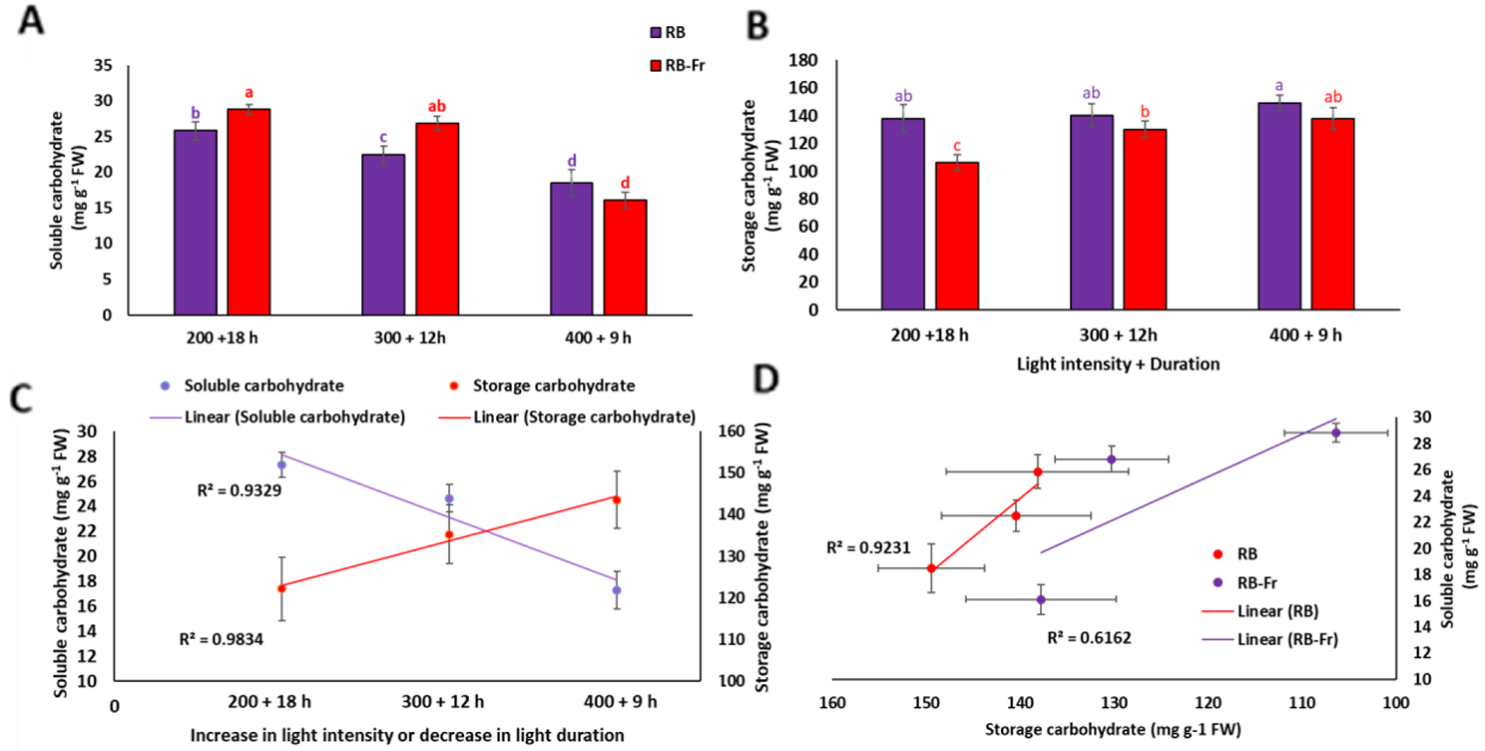
Soluble carbohydrate (**A**) and storage carbohydrate (**B**) of basil plants grown for 25 days at different lighting environments including light intensity of 200, 300, and 400 µmol m^− 2^ s^− 1^ of 70%:30% of red (R): blue (B) light with durations of 18, 12, and 9 h (200 + 18 h, 300 + 12 h, and 400 + 9 h), respectively, resulted in a fixed daily light integral (12.96 mol m^− 2^ d^− 1^) among all light treatment in an indoor controlled environment with (RB + Fr) or without (RB) far red (Fr) light. Columns are the mean value of three replications. Means with the same letters within the groups are not significantly different (*P < 0.05*). Soluble and storage carbohydrate levels in response to increased light intensity or decreased light duration (**C**) and the relationship between soluble and storage carbohydrates in RB and RB + Fr plants (**D**)

Data analysis showed a significant interaction between the light intensity/duration and Fr light for the carbohydrate levels. Soluble carbohydrates had the lowest levels under short light duration and high intensity [LI400/9] with and without Fr, while their amount increased with longer photoperiods and lower light intensities when Fr light was included in the lighting environment [LI200/18 + Fr and LI300/12 + Fr] (Fig. 4A). The level of storage carbohydrates was gradually decreased with longer photoperiods and lower light intensities when Fr light was included in the lighting environment (Fig. 4B).

There was an adverse trend between the soluble and storage carbohydrates levels with an increase in light intensity or decrease in lighting duration in basil leaves (Fig. 4C). The level of soluble carbohydrates showed a decreasing trend. In contrast, storage carbohydrates exhibited an increasing trend by increasing light intensity or decreasing lighting duration. Storage carbohydrate levels decreased when soluble carbohydrates increased (Fig. 4D). There was the same trend when Fr was included or absent in the lighting environment.

### Pigmentation and antioxidant activity of basil plants depend on the duration and intensity of light or the far-red exposure

**Fig. 5:**
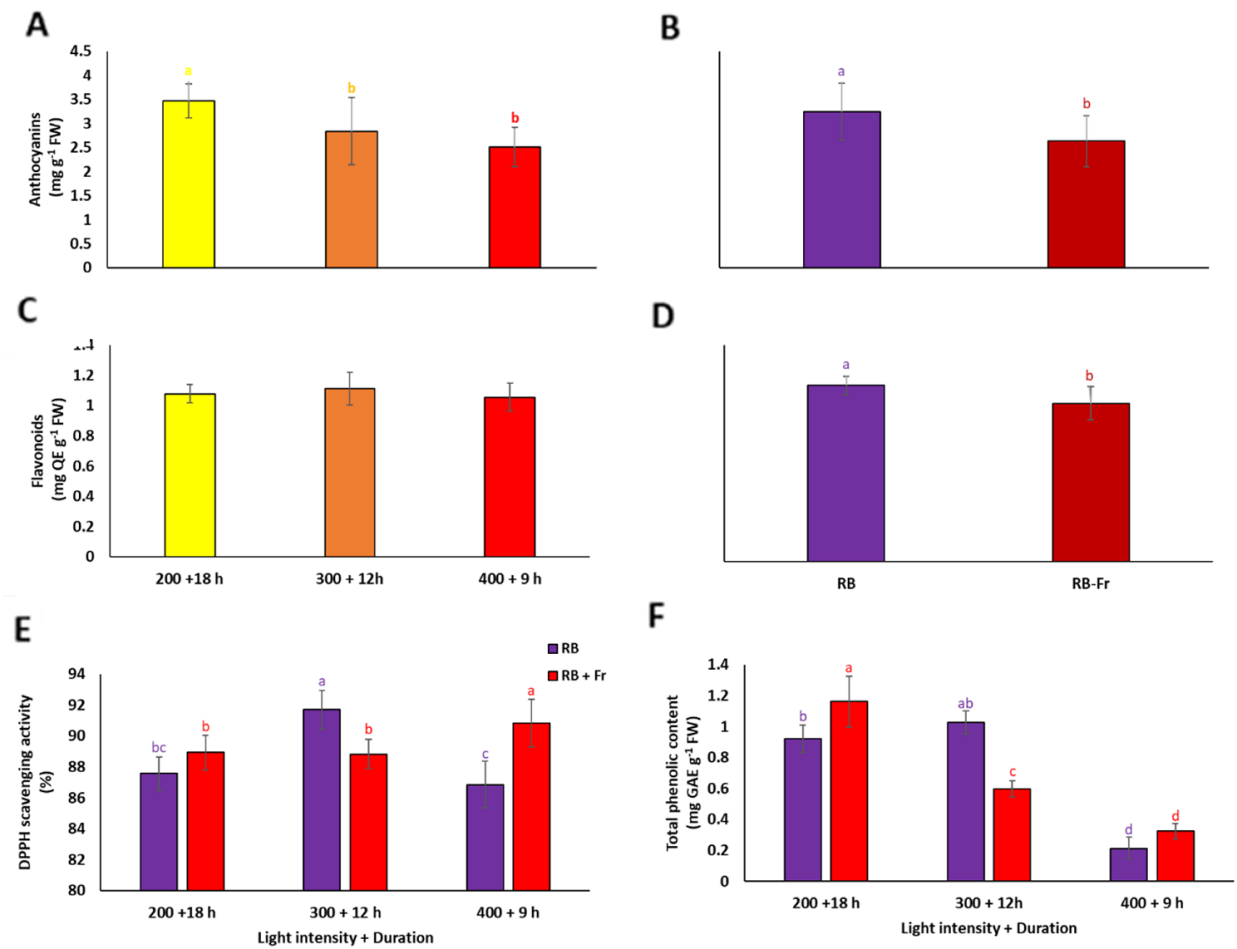
Anthocyanins (**A**, **B**) and flavonoids (**C**, **D**) contents, DPPH scavenging activity (**E**), and total phenolic content (**F**) of basil plants grown for 25 days at different lighting environments including light intensity of 200, 300, and 400 µmol m^− 2^ s^− 1^ of 70%:30% of red (R): blue (B) light with durations of 18, 12, and 9 h (200 + 18 h, 300 + 12 h, and 400 + 9 h), respectively, resulted in a fixed daily light integral (12.96 mol m^− 2^ d^− 1^) among all light treatment in an indoor controlled environment with (RB + Fr) or without (RB) far red (Fr) light. Columns are the mean value of three replications ± SE. Means with the same letters within the groups are not significantly different (*P < 0.05*)

Data analysis showed significant interactions between the light intensity/duration and Fr light regarding DPPH scavenging activity and total phenolic content. No interaction difference was found for the anthocyanins and flavonoids. At the same time, both simple effects of light intensity/duration or Fr light showed significant differences (Fig. 5). The total anthocyanins content of plants demonstrated a significant decrease as the light intensity increased or duration decreased and Fr light negatively impacted anthocyanins (Fig. 5A, B). The level of flavonoids increased on average in plants exposed to Fr light (Fig. 5D). The highest DPPH scavenging activity was detected in plants exposed to LI300/12 without Fr and LI400/9 + Fr (Fig. 5E). The highest total phenolic content was detected in plants under LI200/18 + Fr and LI300/12 (Fig. 5F). The amount of total phenolic content in plants under LI200/18 + Fr was almost six times higher than the LI400/9 with or without Fr.

### Phytohormone levels in basil leaves depend on the lighting environment

**Fig. 6:**
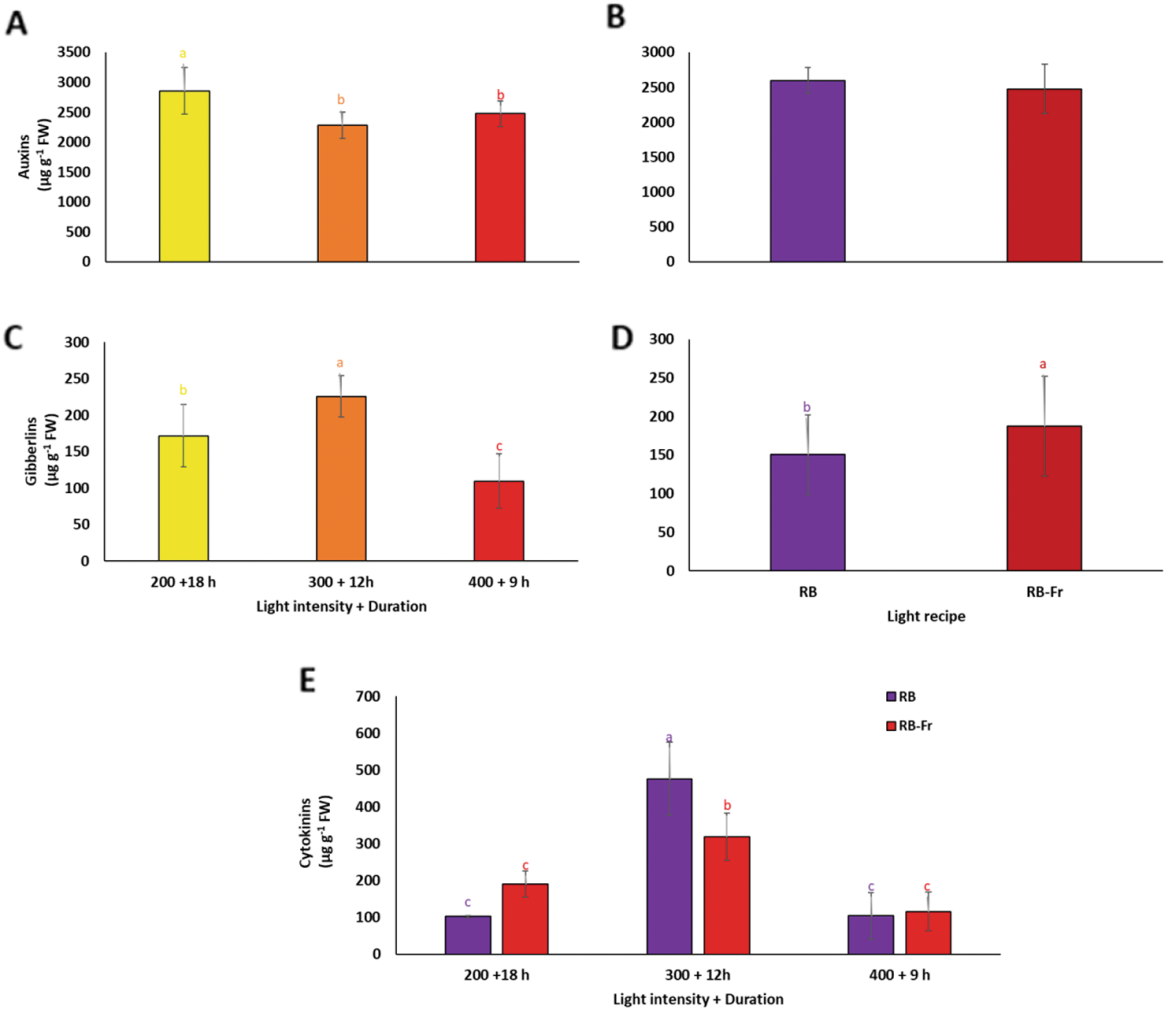
Auxins (**A**, **B**), gibberellins (**C**, **D**), and cytokinins (**E**) levels of basil plants grown for 25 days at different lighting environments including light intensity of 200, 300, and 400 µmol m^− 2^ s^− 1^ of 70%:30% of red (R): blue (B) light with durations of 18, 12, and 9 h (200 + 18 h, 300 + 12 h, and 400 + 9 h), respectively, resulted in a fixed daily light integral (12.96 mol m^− 2^ d^− 1^) among all light treatment in an indoor controlled environment with (RB + Fr) or without (RB) far red (Fr) light. Columns are the mean value of three replications. Means with the same letters within the groups are not significantly different (*P < 0.05*)

Data analysis showed significant interactions between the light intensity/duration and Fr light regarding cytokinin content. In contrast, no interaction was found to be significant for the gibberellins and auxin contents. Both simple effects of light intensity/duration or Fr light showed statistically significant differences for gibberellins, and only the simple effect of light intensity/duration significantly influenced the auxin content (Fig. 6). The auxin level increased in plants exposed to LI200/18. In comparison, the lowest level was detected in plants under LI300/12 and LI400/9 without any significant difference (Fig. 6A).

The highest gibberellins content was detected in plants exposed to LI300/12. In comparison, the lowest gibberellins content was recorded in plants under LI400/9 treatment (Fig. 6C). Exposure to Fr light increased gibberellins content (Fig. 6D). The highest cytokinins content was detected in plants exposed to LI300/12 without Fr. In comparison, their lowest contents were recorded in plants under LI200/18 and LI400/9 with or without Fr (Fig. 6E). On average, the cytokinins levels in plants exposed to LI300/12 without Fr were four times higher than those in other plants.

Correlation analysis among the measured parameters showed significant differences for a positive correlation between soluble carbohydrates and shoot dry weight, root fresh weight, and petiole lengths. In contrast, storage carbohydrates negatively correlated with plant height and petiole length. Total phenolic content was positively correlated with shoot fresh weight, shoot dry weight, and leaf area. Gibberellin content was positively correlated with root fresh weight, root dry weight, and plant height. Shoot fresh weight showed positive correlations with shoot dry weight and leaf area. Positive correlations existed between root fresh weight and root dry weight, plant height, and petiole length. Root dry weight was positively correlated with plant height and petiole length. Finally, plant height and petiole length were positively correlated (Table 1).

**Table 1:** Correlation analysis among the studied traits of basil plants exposed to different lighting environments with fixed daily light integral

Correlations																
Soluble carbohydrate (1)	1.00															
Storage carbohydrate (2)	-0.71	1.00														
Anthocyanin (3)	0.57	-0.16	1.00													
Flavonoids (4)	0.05	0.34	0.63	1.00												
Antioxidant activity (5)	-0.21	-0.09	-0.30	0.08	1.00											
Total phenolic content (6)	0.78	-0.64	0.72	0.36	0.25	1.00										
Auxins (7)	0.38	-0.30	0.73	0.05	-0.61	0.37	1.00									
Gibberelins (8)	0.74	-0.55	0.09	0.07	0.29	0.59	-0.32	1.00								
Cytokinins (9)	0.26	-0.05	0.04	0.50	0.65	0.44	-0.62	0.75	1.00							
Shoot fresh weight (10)	0.75	-0.29	0.74	0.43	0.13	0.86^*^	0.27	0.58	0.49	1.00						
Shoot dry weight (11)	0.82^*^	-0.40	0.73	0.38	0.12	0.89^*^	0.28	0.64	0.49	0.99^**^	1.00					
Root fresh weight (12)	0.86^*^	-0.63	0.32	0.10	0.26	0.78	-0.08	0.95^**^	0.66	0.78	0.83^*^	1.00				
Root dry weight (13)	0.79	-0.58	0.16	-0.03	0.32	0.67	-0.21	0.95^**^	0.67	0.71	0.76	0.98^**^	1.00			
Leaf area (14)	0.72	-0.21	0.8	0.54	0.07	0.84^*^	0.29	0.54	0.50	0.99^**^	0.97^**^	0.73	0.64	1.00		
Plant height (15)	0.71	-0.85^*^	-0.1	-0.36	0.30	0.55	-0.15	0.84^*^	0.41	0.37	0.46	0.83^*^	0.86^*^	0.27	1.00	
Petiole length (16)	0.89^*^	-0.82^*^	0.29	-0.22	0.15	0.76	0.17	0.79	0.32	0.69	0.76	0.91^*^	0.89^*^	0.60	0.89^*^	1.00
	1	2	3	4	5	6	7	8	9	10	11	12	13	14	15	16
.																

* Correlation is significant at the 0.05 level (2-tailed)

** Correlation is significant at the 0.01 level (2-tailed)

## Discussion

In the present study, we investigated the effects of three distinct light conditions, considering a constant DLI, on basil growth and phytochemical compounds within a controlled environment, aiming to determine the optimal condition for maximizing growth and to study the underlying mechanisms. The experimental setup included variations in light intensity and duration with or without Fr light, and the growth parameters of basil plants were assessed accordingly. The studied light treatments significantly influenced plant growth and morphology (Figs. 1 and 2). Longer durations of light exposure with lower intensity, LI200/18 treatment, may allow for a longer duration of photoassimilate production and biomass accumulation in the basil. On the other hand, our data revealed the negative impact of higher light intensity with shorter duration LI400/9 on basil plant growth. At high light intensities, the light-harvesting centers in the plants become saturated, leading to the inefficiency of light utilization and the wastage of excess light energy [37]. This phenomenon is known as photoinhibition. When the light intensity exceeds the capacity of the photosynthetic machinery to process the incoming energy, the excess energy can cause damage to the photosynthetic apparatus, resulting in reduced photosynthetic efficiency and potentially inhibiting plant growth [38, 39]. In contrast, lower light intensities with longer durations allow the plants to balance light absorption and utilization unless continuous light is applied [40]. Although extended exposure to lower light intensities may have less energy provision for the photosynthetic apparatus and may harm photosynthesis, those lighting conditions provide a continuous supply of energy efficiently utilized for photosynthesis and other metabolic processes [41]. This can result in increased biomass accumulation and growth compared to shorter durations of high light intensity, where excess light energy is wasted. However, it is essential to clarify that in this context, the term “lower light” does not refer to light levels that are insufficient to meet the plant’s requirements for photosynthesis.

The current study revealed the negative effect of Fr light on gaining biomass in basil plants (Fig. 1). It has been reported that when the ratio between R and Fr is high, the plants spend their photoassimilate for elongation of stem and petioles in the expense of reduction of their overall shoot biomass [9]. Phytochromes, as R and Fr light receptors, play regulatory control over various biological processes, including photosynthesis, chloroplast formation, synthesis of photosynthetic pigments, and the production of secondary metabolites in the leaves and fruits of plants [42]. Fr light can compete with R light for binding to phytochrome, leading to the conversion of active phytochrome to its inactive form [9, 43]. In some cases, excessive Fr light exposure can result in elongated and spindly growth, as the plants redirect their energy towards stem elongation rather than biomass production, as seen in plants height and petiole length in the current study (Fig. 2B, C). This response is common in plants under dense canopy with limited light. In those circumstances, the upper canopy leaves mostly absorb the R light, and Fr light penetrates and induces stem elongation, a response called ‘shade avoidance response (syndrome)’ [44, 45]. However, it is important to note that the specific response of plants to Fr can vary depending on various factors, including the plant species and genetic traits [46, 47]. Most recently, it has been shown that Fr’s effects on plant growth, physiology, and yield depend on the light intensity plants are exposed to [48]. When plants are exposed to Fr light, several physiological and molecular responses occur that can affect the plant’s developmental and growth processes, like leaf expansion. In environments with high Fr levels, there would be a change in biomass allocation to different plants’ organs [5, 49, 50].

**Fig. 7:**
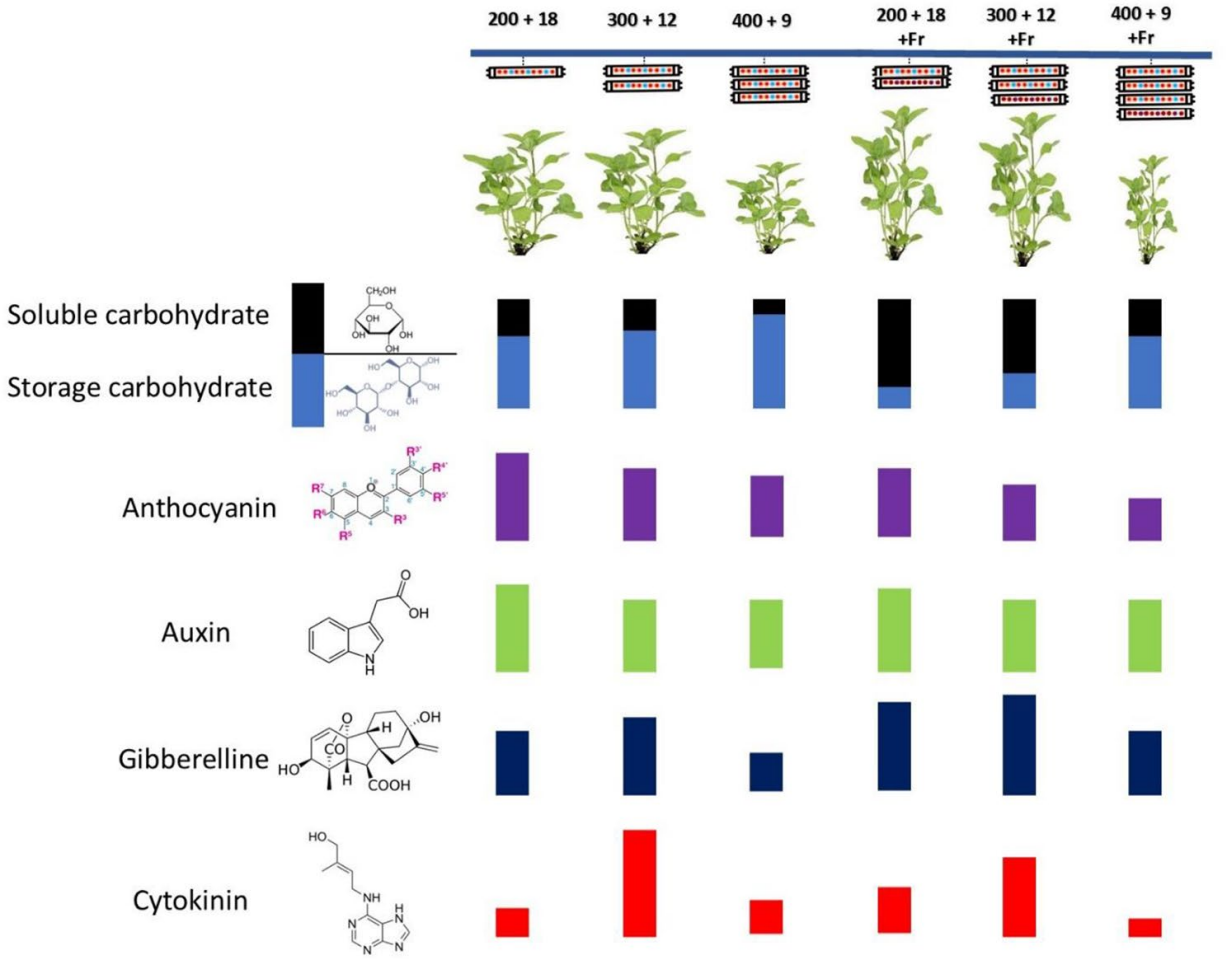
A schematic diagram summarizing basil plants’ growth, phytochemical, and phytohormonal levels in response to different lighting strategies. Basil plants were grown for 25 days at different lighting environments including light intensity of 200, 300, and 400 µmol m^− 2^ s^− 1^ of 70%:30% of R: B light with durations of 18, 12, and 9 h (200 + 18, 300 + 12, and 400 + 9), respectively, resulted in a fixed daily light integral (12.96 mol m^− 2^ d^− 1^) among all light treatment in an indoor controlled environment with or without far red (Fr) light. Growth is limited by exposure to 400 + 9 with or without Fr probably due to low levels of growth promoting phytohormones. Adding Fr to lighting environment resulted in an increase in the soluble to storage carbohydrate levels ratio. Furthermore, anthocyanin level was decreased by 400 + 9 lighting recipe

The observation of a decrease in leaf area when Fr light was included in the lighting environment with R: B lights in our study could be attributed to several factors. An alternative explanation is the competitive binding of Fr light with R light to phytochrome. In the presence of Fr light, the absorption of R light by phytochrome is reduced, leading to a shift in the phytochrome equilibrium towards the inactive form (Pr). This shift can result in altered signaling pathways and changes in plant physiology, including reduced leaf expansion and growth. Additionally, the interaction between Fr light and the R: B light spectrum may disrupt the balance of different photoreceptors involved in leaf development. B light, for instance, promotes leaf expansion and development [51, 52]. As a general morphological response to Fr, plants elongate their stems, petioles, and internodes to seek more light. However, the type of shade response depends on the species’ vulnerability to shading conditions [53–55]. FR light induces growth in shade-tolerant plants by increasing leaf area and light interception, promoting photosynthesis and biomass gaining [56]. Conversely, exposure to low intensities of Fr may induce leaf expansion and growth to some extent in species that are sensitive to shade and show strong SAS. However, high Fr intensity exposure would result in a low R/Fr ratio and decrease PPS; in such circumstances, shade-sensitive species allocate their biomass more to the stem and petiole (for elongation) at the expense of leaf area, leading to lower photosynthesis and biomass [56]. This was in line with our findings on the positive effects of Fr light on the elongation of stem and petiole while negatively affecting the overall basil biomass (Figs. 1, 2 and 7).

Including Fr light alongside R and B lights may interfere with the signaling pathways associated with B light perception, potentially leading to reduced leaf area. Furthermore, the negative effect of Fr on leaf area under R and B lights could be related to its impact on photosynthesis and biomass accumulation. However, extending duration in the absence of Fr positively influenced leaf area and petiole growth as substantial characteristics for basil growth related to the increased photosynthesis parameters under longer light intensity, as we can see the soluble carbohydrates increased by the longest light duration. As said before, in dense canopies, where R light is predominantly absorbed in the higher portion of the canopy, Fr light becomes more prevalent. This shift in light composition triggers a series of responses, including reduced photosynthetic efficiency and a competitive signal for stem elongation, enabling plants to capture more light [44]. This response provides a means to modulate plant architecture, optimize light capture, and potentially enhance productivity in plant factory settings. Notably, the interactive effect of R and B light emission with lower light intensity can potentially increase the accumulation of soluble carbohydrates in plants, resulting in better taste [57, 58]. B light is absorbed by chlorophyll and other photoreceptors, stimulating the light-dependent reactions of photosynthesis [59, 60]. This results in increased production of ATP and NADPH, which provide the energy and reduce the power needed for carbohydrate synthesis. Moreover, mixed B and R light influences stomatal opening and closure [61]. B light can promote stomatal opening by activating specific phototropin receptors, increasing carbon dioxide uptake for photosynthesis [62]. This elevated carbon dioxide availability can enhance the production of carbohydrates. Furthermore, R light directly influences leaf carbohydrate accumulation [56]. Carbohydrates are an energy source for hormonal regulation, cell division, elongation, and differentiation [25]. Increased carbohydrate availability can promote overall plant growth and biomass accumulation. These carbohydrates are crucial in cell enlargement and turgor pressure maintenance [7]. With higher carbohydrate levels, plants have more resources for leaf expansion, leading to larger leaf sizes and increased leaf area [63]. In the current study, Fr light increased soluble and reduced storage carbohydrates. Fr light has been shown to promote assimilating production, including soluble carbohydrates [64]. It can stimulate the activity of key enzymes involved in carbohydrate metabolism, such as sucrose synthase and invertase, leading to increased synthesis and accumulation of soluble carbohydrates [65, 66]. Our finding in the current study by applying Fr on accumulated carbohydrate content aligns with previous research in the basil plant [66]. The level of storage carbohydrates increased in plants exposed to higher light intensity/lower light duration and vice versa (Fig. 4). In accordance, it has been reported that when plants are exposed to high light intensities, they supply energy exceeds its demands, under such circumstances, the plants tend to reserve the carbohydrates to make efficient use of energy and to prevent stress on its photosynthesis apparatus [67].

Our treatments also evidenced induced secondary metabolite by R/B light. It has been reported that quality of growing light environment has a great impact on metabolite composition of leaf [68]. B light has been found to promote plants’ biosynthesis of anthocyanins and flavonoids [66]. It activates specific photoreceptors called cryptochromes, which play a role in regulating various developmental processes. When plants are exposed to B light, the expression of genes like ABA-regulating genes is upregulated, increasing anthocyanin and flavonoid content (Fig. 5A, B) [69]. R light promotes converting the inactive form of phytochrome (Pr) to the active form (Pfr). The active form of phytochrome can activate transcription factors that regulate gene expression in anthocyanin biosynthesis [70]. However, in some cases, R light is not an effective inducer of anthocyanin production, implying that light intensity and quality may induce different signal transudation [71]. In our experiment, the duration and intensity changes did not significantly affect anthocyanin content. However, Fr light reduced both metabolites. Paralleled with this evidence, by converting the Pfr back to the Pr, Fr light reduces the overall synthesis of anthocyanins and flavonoids [46], as observed in our results (Fig. 5A, B). On the other hand, B and R light increases anthocyanin and flavonoid levels. By promoting the biosynthesis of these antioxidant compounds, B and R lights can increase DPPH scavenging activity. The cumulative effect of these components has been observed in DPPH scavenging activity, which has been shown under medium light intensity and duration (Fig. 5C). However, the highest light intensity and lowest duration represented minimum phenolic content. Phenolic compounds are susceptible to degradation under high light intensity and temperature [72]. Exposure to intense light can lead to the breakdown or oxidation of phenolic compounds, reducing their content [73]. This degradation can occur through various mechanisms, including direct photochemical reactions or the generation of ROS [6, 37]. Plants allocate resources based on their physiological needs. When exposed to high light intensity, plants may prioritize allocating resources towards processes such as photosynthesis, growth, and defense against oxidative stress. This allocation may come at the expense of phenolic compound production, leading to a decrease in phenolic content (Fig. 5D). In our study, storage carbohydrates negatively correlated to the total phenolic content of the plant (Table 1). In accordance, it has been reported that high phenolic content induces starch degradation and increases the solubility of the starch [74].

Red and B light can affect the synthesis and distribution of plant hormones such as auxins, cytokinins, and gibberellins [37, 74]. These phytohormones play vital roles in regulating plant growth and development, including carbohydrate partitioning and allocation. B light has been found to stimulate auxin production in various plant tissues [75]. B light-induced auxin production is often associated with phototropism, which is the directional growth response of plant organs towards light [76, 77]. R light can also influence auxin production in plants. It is perceived by the phytochrome photoreceptors, which can regulate the expression of genes involved in auxin biosynthesis and signaling pathways [78, 79]. Plants may increase auxin production under low light intensity to promote elongation and growth in search of more light [80]. Resource allocation for plant elongation under low light intensity can lead to a shift in hormone balance. Usually, there is an inverse relationship between auxin levels and cytokinin production, which also affects gibberellin levels (Fig. 6C, E). Despite low auxin levels under LI300/12, accumulation of cytokinins prevented the reduction of leaf area, causing observation of similar leaf area between LI300/12 and LI200/18 (Figs. 2A and 7). Although auxin levels were reduced under LI300/12, high gibberellin levels led to similar petiole growth as in the LI200/18 (Figs. 2C, 6C and 7). The decrease in auxin levels under medium light intensity and duration indicates a possible light-dependent modulation of auxin biosynthesis or transport. This reduction may be associated with alterations in plant growth and development, such as changes in leaf area and petiole growth. Therefore, cytokinin accumulation in plants exposed to LI300/12, suggests a compensatory path to maintain adequate growth and physiological processes. Cytokinins are crucial in promoting growth, mainly when auxin availability is limited. This indicates a complicated molecular pathway, potential crosstalk, and functional redundancy between auxin and cytokinins in regulating plant growth under different light conditions.

Complex molecular interactions have been reported between auxin, gibberellins, and cytokinins for the induction of leaf and stem growth [80]. Several positive and negative molecular interactions have been elucidated among different stages (biosynthesis, signal transduction, and response) of plant response to endogenous phytohormones [80]. For instance, the DELLA protein, the key negative regulator of gibberellin signaling, interacts with other phytohormones, determining SAS and other plant growth responses [80]. Based on the result of the present study, the comparable petiole growth observed with gibberellin accumulation under LI200/18, similar to the phenotype detected in plants under LI400/9 + Fr, suggests that gibberellins may play a prominent role in promoting elongation and development of petioles [80], even in the absence of high auxin levels. Accordingly, a strong positive correlation was found between gibberellins and plant height of basil plants (Table 1).

The interplay between cytokinin and auxin ratios is also paramount in regulating plant growth and development. Notably, an elevated auxin-to-cytokinin ratio has been shown to stimulate shoot apical meristem (SAM) activity, resulting in a more significant number of leaf primordia and subsequent leaf formation. During the initiation of leaf primordia, the ERECTA family receptors play a dual role, and they suppress the action of cytokinins, inhibiting their effects. On the other hand, they stimulate the development of leaf primordia by upregulating the expression of PIN-FORMED 1 (PIN1), which results in enhanced polar transport of auxin [81]. Auxin is involved in various aspects of plant growth and development, including leaf initiation and development. It helps form and position leaf primordia and the initial stages of leaf development. Auxin gradients within the growing SAM contribute to the initiation of new leaf primordia [82].

## Conclusion and future prospective

The current experiment supports using low or medium light intensity with a longer duration for producing basil plants in a controlled environment. Low light intensity with the associated changes in the plant’s biological and metabolic pathways diverting energy and resources towards vegetative growth. The findings also support higher antioxidant properties within low or medium light intensity with a longer duration. Alteration in hormonal production and accumulation caused more growth and shade avoidance responses in basil plants. The findings suggest balancing light intensity and duration is crucial for basil growth in a controlled environment. Such optimized light conditions can lead to production of more nutritious produce as well as sustainable and cost-effective cultivation practices. Given the complexity of determining optimal light conditions for basil cultivation in controlled environments, further research is essential to elucidate the underlying mechanisms. This includes investigating specific light intensities, durations, and spectral compositions that yield optimal results across varying conditions. Areas requiring further exploration encompass but are not limited to: (**I**) analyzing basil’s growth and metabolite profiling under different full spectrum light qualities, including white and green light, to ascertain whether longer light durations remain advantageous. (ii) investigating the relationship between proper light intensity for basil growth and the duration and quality of light, particularly when utilizing varying compositions of R and B lights. (iii) exploring the impact of non-continuous light durations, such as different light/dark cycles within 24 h, alongside varying light intensities and qualities, while maintaining a consistent daily light integral (DLI) on basil growth and metabolite profile. (iv) examining the effects of night interruption using diverse light qualities and intensities while maintaining a consistent DLI on basil growth and metabolite production, and (v) exploring the molecular mechanisms involved in plant responses to lighting strategies. Since having fixed DLI means a change in circadian clocks, we suggest studying the genes involved in both the circadian clock and light responses of the plants.

## Data Availability

Data would be available in request from Sasan Aliniaeifard.
